# Supplementary material for: Accelerated discovery of highly active enzyme nanohybrids with parallelized Bayesian optimization in hybrid space
Source: Nat Commun. 2026 Mar 7;17:3634. doi: 10.1038/s41467-026-70251-3 (PMC13096166; doi:10.1038/s41467-026-70251-3)
Supplement: Supplementary file 1 — Supplementary Information [file 41467_2026_70251_MOESM1_ESM.pdf]

Supplementary Information

Accelerated discovery of highly active enzyme nanohybrids with parallelized Bayesian optimization in hybrid space

Yu Liu[1,2,#], Haoyang Hu[3,#], Yueheng Han[3], Jia Song Deon Chon[1], Chin Lee Lo[1], Zhixuan Chen[1], Zheng Zhang[1], Zhihong Yuan[3,*], Jun Ge[1,4,*]

[1] Key Lab for Industrial Biocatalysis, Ministry of Education, Department of Chemical Engineering, Tsinghua University, Beijing 100084, China

[2] Synthera Biotechnology Co., Ltd.; Shenzhen, 518000, China

[3] The State Key Laboratory of Chemical Engineering and Low-carbon Technology, Department of Chemical Engineering, Tsinghua University, Beijing 100084, China

[4] State Key Laboratory of Green Biomanufacturing, Beijing 100084, China

* Corresponding authors: junge@mail.tsinghua.edu.cn; zhihongyuan@mail.tsinghua.edu.cn

[#] These authors contributed equally to this work.

Materials

Supplementary Table 1. Information of the substances used.

Substance	Producer	CAS	Purity
$Zn(CH_3COO)_2{\cdot}2H_2O$	Sigma-Aldrich	5970-45-6	99%
$Zn(C_3H_5O_3)_2$	Macklin	16039-53-5	98%
$Zn(H_2PO_4)_2{\cdot}2H_2O$	Sinopharm Chemical	13598-37-3	CP
$Zn(NO_3)_2{\cdot}6H_2O$	Sigma-Aldrich	10196-18-6	99%
$ZnSO_4$	BidePharmatech	7733-02-0	99%
$ZnCl_2$	Macklin	7646-85-7	99%
$ZnBr_2$	BidePharmatech	7699-45-8	99.24%
Imidazole	BidePharmatech	288-32-4	99.58%
2-Methylimidazole	Acros Organics	693-98-1	99%
2-Ethylimidazole	BidePharmatech	1072-62-4	98%
4(or 5)-Methylimidazole	BidePharmatech	822-36-6	99.98%
2,4(or 2,5)-Dimethylimidazole	BidePharmatech	930-62-1	99.99%
4,5-Dimethylimidazole	Accela	2302-39-8	97%
2,4,5-Trimethylimidazole	BidePharmatech	822-90-2	95%
1-Methoxymethyl-1H-imidazole	BidePharmatech	20075-26-7	98%
Purine	BidePharmatech	120-73-0	99.71%
Histamine	BidePharmatech	51-45-6	99.56%
Histidine	BidePharmatech	71-00-1	98%
2-Propylimidazole	BidePharmatech	50995-95-4	98%
1H-Imidazol-2-ylmethanol	BidePharmatech	3724-26-3	97%
2-Imidazolidinone	BidePharmatech	120-93-4	98%
2-Mercaptoimidazole	BidePharmatech	872-35-5	97%
Thiazole	BidePharmatech	288-47-1	98%
2-Methylthiazole	BidePharmatech	3581-87-1	99.87%

Supplementary Note 1: Five variables. 7 zinc salts, 17 ligands, [0, 0.01, 1] concentration with 0.01 as step size (100 each), [30, 5, 120] min for reaction time (19), sum= 22,610,000 experiments

Supplementary Table 2. Precursors abbreviation and solubility.

No.	Substance	Abbreviation	M_w (g mol^{-1})	Lower limits (M)	Upper limits (M)
Z1	$Zn(NO_3)_2·6H_2O$	$Zn(NO_3)_2$	297.49	0.001	10.000
Z2	$ZnCl_2$	$ZnCl_2$	136.30	2.000	10.000
Z3	$Zn(CH_3COO)_2·2H_2O$	$Zn(Ac)_2$	219.51	0.001	0.800
Z4	$ZnSO_4$	$ZnSO_4$	161.47	0.001	4.000
Z5	$Zn(H_2PO_4)_2·2H_2O$	$Zn(H_2PO_4)_2$	295.44	0.001	0.800
Z6	$Zn(C_3H_5O_3)_2$	$Zn(C_3H_5O_3)_2$	243.55	0.001	0.120
Z7	$ZnBr_2$	$ZnBr_2$	225.22	0.001	10.000
L1	Imidazole	IM	68.08	0.001	10.000
L2	2-Methylimidazole	2-mIM	82.104	0.001	4.000
L3	2-Ethylimidazole	2-eIM	96.13	0.001	10.000
L4	2-Propylimidazole	2-pIM	110.16	0.001	5.000
L5	4(or 5)-Methylimidazole	4-mIM	82.1	0.001	10.000
L6	2,4(or 2,5)-Dimethylimidazole	2,4-dmIM	96.13	0.001	10.000
L7	4,5-Dimethylimidazole	4,5-dmIM	96.13	0.001	1.000
L8	2,4,5-Trimethylimidazole	2,4,5-tmIM	110.16	0.001	10.000
L9	1H-Imidazol-2-ylmethanol	2-mtIM	98.1	0.001	10.000
L10	1-Methoxymethyl-1H-imidazole	1-mOmIM	112.13	0.001	9.810
L11	2-Mercaptoimidazole	2-tIM	100.14	0.001	0.600
L12	Purine	PR	120.11	0.001	1.250
L13	Histamine	HTM	111.15	0.001	10.000
L14	2-Imidazolidinone	2-IMdn	86.09	0.001	10.000
L15	Thiazole	TZ	85.13	0.001	14.090
L16	2-Methylthiazole	2-mTZ	99.15	0.001	11.090
L17	Histidine	His	155.15	0.001	0.250

Supplementary Tables

Supplementary Table 3. Reaction conditions of immobilized GOx biocatalysts with high activity.

No.	Zinc type	Zn concentration (M)	Ligand type	Ligand concentration (M)	Molar ratio of ligand/Zn	Reaction time (min)	Activity recovery (%)
ZIF	$Zn(NO_3)_2$	0.31	2-mIM	1.25	40.3	30	5.2±0.1
A1	$Zn(CH_3COO)_2$	0.155	2,4-dmIM	0.625	40.3	30	86.7±1.6
A2	$Zn(CH_3COO)_2$	0.0851	2,4-dmIM	0.5273	62.0	30	73.9±6.8
A3	$Zn(CH_3COO)_2$	0.2595	2,4-dmIM	0.01	0.4	110	74.6±9.0
A4	$Zn(NO_3)_2$	1.0616	2,4-dmIM	0.5358	5.1	30	82.0±2.6
A5	$ZnBr_2$	1.0616	2,4-dmIM	0.5358	5.1	30	89.7±3.0
A6	$Zn(CH_3COO)_2$	0.6134	PR	0.1658	2.7	30	86.4±5.0
A7	$Zn(CH_3COO)_2$	0.2601	2-eIM	0.01	0.4	40	75.2±10.5
A8	$Zn(CH_3COO)_2$	0.6333	HTM	0.0341	0.5	35	88.4±11.1
B1	$Zn(CH_3COO)_2$	0.6372	2-tIM	0.0426	0.7	30	95.6±1.4
B2	$Zn(CH_3COO)_2$	0.5975	2-tIM	0.0515	0.9	30	101.4±1.2
B3	$Zn(CH_3COO)_2$	0.6571	2-tIM	0.0693	1.1	120	108.8±0.3
B4	$Zn(CH_3COO)_2$	0.6174	2-tIM	0.0752	1.2	30	114.0±1.9
B5	$Zn(CH_3COO)_2$	0.6452	2-tIM	0.0812	1.3	50	105.0±0.4
B6	$Zn(CH_3COO)_2$	0.5817	2-tIM	0.0812	1.4	30	116.4±0.6
B7	$Zn(CH_3COO)_2$	0.5261	2-tIM	0.0989	1.9	45	110.0±0.3

Supplementary Table 4. Reaction conditions of immobilized CAT biocatalysts with high activity.

No.	Zinc type	Zn concentration (M)	Ligand type	Ligand concentration (M)	Molar ratio of ligand/Zn	Reaction time (min)	Activity recovery (%)
ZIF	$Zn(NO_3)_2$	0.3100	2-mIM	1.2500	40.3	30	57.0±1.8
C1	$Zn(CH_3COO)_2$	0.7127	HTM	0.1487	2.1	30	94.8±10.4
C2	$Zn(CH_3COO)_2$	0.5817	HTM	0.1137	2.0	30	97.1±7.3
C3	$Zn(CH_3COO)_2$	0.6094	HTM	0.0908	1.5	75	93.2±9.0
C4	$Zn(CH_3COO)_2$	0.7563	HTM	0.1113	1.5	45	93.9±7.9
C5	$Zn(NO_3)_2$	1.0616	2,4-dmIM	0.5358	5.0	30	87.2±2.2
C6	$ZnBr_2$	1.0616	2,4-dmIM	0.5358	5.0	30	85.7±1.8
C7	$Zn(NO_3)_2$	1.1646	HTM	0.0100	0.1	30	82.6±4.5
C8	$Zn(CH_3COO)_2$	0.1013	2,4-dmIM	0.2108	20.8	35	83.1±3.3
C9	$Zn(CH_3COO)_2$	0.1550	2,4-dmIM	0.6250	40.3	30	81.0±6.4
C10	$Zn(NO_3)_2$	0.7128	HTM	0.0510	0.7	105	76.6±6.4
C11	$Zn(NO_3)_2$	1.4156	4,5-dmIM	0.0100	0.1	30	74.2±8.0

Supplementary Table 5. Reaction conditions of immobilized CALB biocatalysts with high activity.

No.	Zinc type	Zn concentration (M)	Ligand type	Ligand concentration (M)	Molar ratio of ligand/Zn	Reaction time (min)	Activity recovery (%)
ZIF	$Zn(NO_3)_2$	0.3100	2-mIM	1.2500	40.3	30	1.4±1.0
D1	$ZnBr_2$	2.1686	2,4-dmIM	0.7128	3.3	110	65.5±5.4
D2	$ZnBr_2$	2.0682	2,4-dmIM	0.8634	4.2	90	73.8±5.1
D3	$ZnBr_2$	1.8172	2,4-dmIM	0.9136	5.0	85	70.1±6.1
D4	$ZnBr_2$	1.0616	2,4-dmIM	0.5358	5.0	30	70.5±9.0
D5	$ZnBr_2$	1.8674	2,4-dmIM	1.1144	6.0	95	68.7±2.9
D6	$ZnBr_2$	1.0642	2,4-dmIM	0.8634	8.1	45	70.8±2.4
E1	$Zn(NO_3)_2$	1.0642	2,4-dmIM	0.7128	6.7	75	72.4±2.3
E2	$Zn(NO_3)_2$	0.9136	2,4-dmIM	1.0140	11.1	80	77.4±2.9
E3	$Zn(NO_3)_2$	0.7128	2,4-dmIM	0.9638	13.5	75	76.5±1.4
E4	$Zn(NO_3)_2$	0.9638	2,4-dmIM	1.3152	13.6	75	75.1±2.6
E5	$Zn(NO_3)_2$	0.3614	2,4-dmIM	0.5120	14.2	30	72.4±2.1
E6	$Zn(NO_3)_2$	0.5120	2,4-dmIM	0.8634	16.9	80	71.3±2.9

Supplementary Table 6. Comparison of the effectiveness of transfer learning between PHBO and control baseline: in the case of immobilized CAT biocatalysts.

Group	Zinc type	Zn concentration (M)	Ligand type	Ligand concentration (M)	Molar ratio of ligand/Zn	Reaction time (min)	Activity recovery (%)
PHBO	$Zn(Ac)_2$	0.1550	2,4-dmIM	0.6250	40.3	30	85.7±6.1
	$Zn(NO_3)_2$	1.0616	2,4-dmIM	0.5358	5.0	30	90.8±1.6
	$ZnBr_2$	1.0616	2,4-dmIM	0.5358	5.0	30	87.0±1.5
	$Zn(Ac)_2$	0.6134	PR	0.1658	2.7	30	79.7±1.3
	$Zn(Ac)_2$	0.2601	2-eIM	0.0100	0.4	30	54.9±2.2
	$Zn(Ac)_2$	0.6333	HTM	0.0341	0.5	30	85.8±1.2
	$Zn(Ac)_2$	0.5817	2-tIM	0.0814	1.4	30	47.9±0.3
	$Zn(Ac)_2$	0.5817	2-tIM	0.2327	4.0	30	38.7±0.8
Control baseline	$ZnSO_4$	3.9591	2-mtIM	6.2296	15.7	50	14.7±0.5
	$Zn(C_3H_5O_3)_2$	0.0039	2-IMdn	3.4604	8872.8	105	1.9±0.5
	$ZnSO_4$	1.9194	2,4-dmIM	8.8332	46.0	55	0.0±0.0
	$Zn(H_2PO_4)_2$	0.2756	2-eIM	0.3678	13.3	40	54.7±2.3
	$Zn(Ac)_2$	0.0959	2-tIM	0.0674	7.0	80	0.6±0.6
	$Zn(Ac)_2$	0.6512	PR	0.5681	8.7	65	71.7±1.9
	$Zn(NO_3)_2$	2.2209	2-IMdn	5.5182	24.8	40	38.1±1.3
	$Zn(Ac)_2$	0.6112	4-mIM	2.9172	47.7	120	48.3±0.6

Supplementary Note 2: The control baseline experimental group was recommended without any prior knowledge. The conditions extensively explored the entire reaction space, with precursor concentrations reaching up to 8.8 M and a ligand-to-zinc ratio of 8872.8. The exploration efficiency was relatively low, resulting in an average AR of only 28.74%. In contrast, PHBO made recommendations based on knowledge extracted from the GOx experimental data. The conditions explored more of the low precursor concentration and low ligand-to-zinc ratio space, leading to an average AR of 71.31%.

Supplementary Table 7. Comparison of the effectiveness of transfer learning between PHBO and control baseline: in the case of immobilized CALB biocatalysts.

Group	Zinc type	Zn concentration (M)	Ligand type	Ligand concentration (M)	Molar ratio of ligand/Zn	Reaction time (min)	Activity recovery (%)
PHBO	$Zn(NO_3)_2$	1.0616	2,4-dmIM	0.5358	5.0	30	51.4±8.5
	$ZnBr_2$	1.0616	2,4-dmIM	0.5358	5.0	30	57.2±2.5
	$Zn(Ac)_2$	0.6134	PR	0.1658	2.7	30	32.3±1.5
	$Zn(Ac)_2$	0.5817	2-tIM	0.0814	1.4	30	17.7±0.5
	$Zn(Ac)_2$	0.5817	2-tIM	0.2327	4.0	30	31.9±1.1
	$Zn(Ac)_2$	0.6174	2-tIM	0.0752	1.2	30	13.7±0.8
	$Zn(Ac)_2$	0.5817	2-tIM	0.4654	8.0	30	8.9±0.5
	$Zn(Ac)_2$	0.2067	2,4-dmIM	0.8333	40.3	30	14.8±1.3
Control baseline	$ZnBr_2$	8.1262	2-tIM	0.3613	0.4	50	18.3±1.7
	$ZnSO_4$	0.1058	2-tIM	0.0531	5.0	85	0.0±9.0
	$Zn(H_2PO_4)_2$	0.3748	2-mIM	1.1350	30.3	30	1.0±0.9
	$Zn(NO_3)_2$	0.1811	PR	0.8154	45.0	100	11.2±0.7
	$ZnBr_2$	9.3371	2,4-dmIM	1.6148	1.7	65	0.0±0.0
	$Zn(C_3H_5O_3)_2$	0.0214	1-mOmIM	0.525	245.3	70	0.0±0.0
	$Zn(Ac)_2$	0.7879	TZ	4.9201	62.4	45	0.0±0.0
	$ZnSO_4$	1.0340	4-mIM	7.6677	74.2	60	4.7±0.9

Supplementary Note 3: Similar results can be observed for CALB as well. The control baseline experimental group explored the reaction space more widely, with a ligand-to-zinc ratio ranging from 0.4 to 245.3. CALB faced difficulty in forming nanohybrids and relatively low AR. In this case, 3 out of 8 conditions recommended by control baseline failed to form participates. The average AR generated by PHBO and control baseline were 28.49% and 4.39%, respectively.

Supplementary Table 8. Comparison of prediction by PHBO and real experimental results.

Enzyme type	Zinc type	Zn concentration (M)	Ligand type	Ligand concentration (M)	Reaction time (min)	Predicted activity recovery (%)	Real activity recovery (%)
GOx	$Zn(CH_3COO)_2$	0.5817	2-tIM	0.0081	30	34.2±39.2	0.3±0.1
GOx	$Zn(CH_3COO)_2$	0.0581	2-tIM	0.0814	30	0.0±59.8	0.2±0.0
GOx	$Zn(CH_3COO)_2$	0.0407	2-tIM	0.0814	30	0.0±60.3	0.1±0.0
GOx	$Zn(CH_3COO)_2$	0.0204	2-tIM	0.0814	30	0.0±60.9	0.0±0.0
GOx	$Zn(CH_3COO)_2$	0.0136	2-tIM	0.0814	30	0.3±61.0	0.0±0.0
GOx	$Zn(CH_3COO)_2$	0.0102	2-tIM	0.0814	30	0.8±61.1	0.1±0.0
CALB	$Zn(NO_3)_2$	0.9136	2,4-dmIM	0.0091	80	12.7±6.4	4.5±0.3
CALB	$Zn(NO_3)_2$	0.9136	2,4-dmIM	0.0914	80	31.6±6.0	48.8±1.7
CALB	$Zn(NO_3)_2$	0.9136	2,4-dmIM	0.3654	80	60.7±3.7	68.6±2.6
CALB	$Zn(NO_3)_2$	0.9136	2,4-dmIM	0.7309	80	68.6±6.0	78.4±2.3
CALB	$Zn(NO_3)_2$	0.9136	2,4-dmIM	1.8272	80	38.7±48.3	29.1±1.0
CAT	$Zn(CH_3COO)_2$	0.5817	HTM	0.0114	30	83.6±21.3	82.8±3.9
CAT	$Zn(CH_3COO)_2$	0.5817	HTM	0.0569	30	96.7±12.8	75.4±1.8
CAT	$Zn(CH_3COO)_2$	0.5817	HTM	0.2327	30	72.0±30.8	83.6±1.1

Supplementary Table 9. Enzymatic activity of GOx, CAT, and CALB both in the free form and immobilized in the respectively best carrier.

	Molar extinction coefficient (M^{-1} cm^{-1})	Enzyme activity (U)		Enzyme concentration in the reaction (μg mL^{-1})	Encapsualtion efficiency (%)	Relative activity (%)	Activity recovery (%)
		Free enzyme	Immobilized in best carrier				
GOx	33000 (oxidized ABTS)[1]	0.118±0.001	0.119±0.002	0.064	100.00	100.86	100.86
CAT	43.6 (H_2O_2)[2]	5.614±0.106	4.986±0.082	0.283	99.00	88.82	89.91
CALB	17500 (p-nitrophenol)[3]	6.150±0.130	4.946±0.068	1.475	100.00	80.43	87.70

Supplementary Note 4:

GOx: 3 mL reaction system, substrate was 47 mM glucose, containing 45 μg mL^{-1} HRP and 45 μg mL^{-1} ABTS. The final concentration of free enzyme and immobilized enzyme was 0.064 μg mL^{-1}. The degradation of glucose was determined by the change of absorbance at 415 nm within 15s of the production of oxidized ABTS, which was the activity of GOx. The amount of enzyme that decomposed 1 micromole of glucose and generated 1 micromole of oxidized ABTS per minute was defined as 1U.

CAT: 3 mL reaction system, substrate was 9.8 mM hydrogen peroxide, the final concentration of free enzyme and immobilized enzyme was 0.283 μg mL^{-1} . The activity of CAT was determined by the decrease of absorbance at 240 nm (characteristic absorption peak of hydrogen peroxide) within 15 s. The amount of enzyme that decomposed 1 micromole of hydrogen peroxide per minute was defined as 1U.

CALB: 3 mL reaction system, substrate was 0.90 μg mL^{-1} p-NPB, the final concentration of free enzyme and immobilized enzyme was 1.475 μg mL^{-1} . The degradation of p-NPB was determined by the change of absorbance at 405 nm within 15 s of the production of p-nitrophenol, which was the activity of CALB. The amount of enzyme that decomposed 1 micromole of p-NPB and generated 1 micromole of p-nitrophenol per minute was defined as 1U.

Supplementary Figures

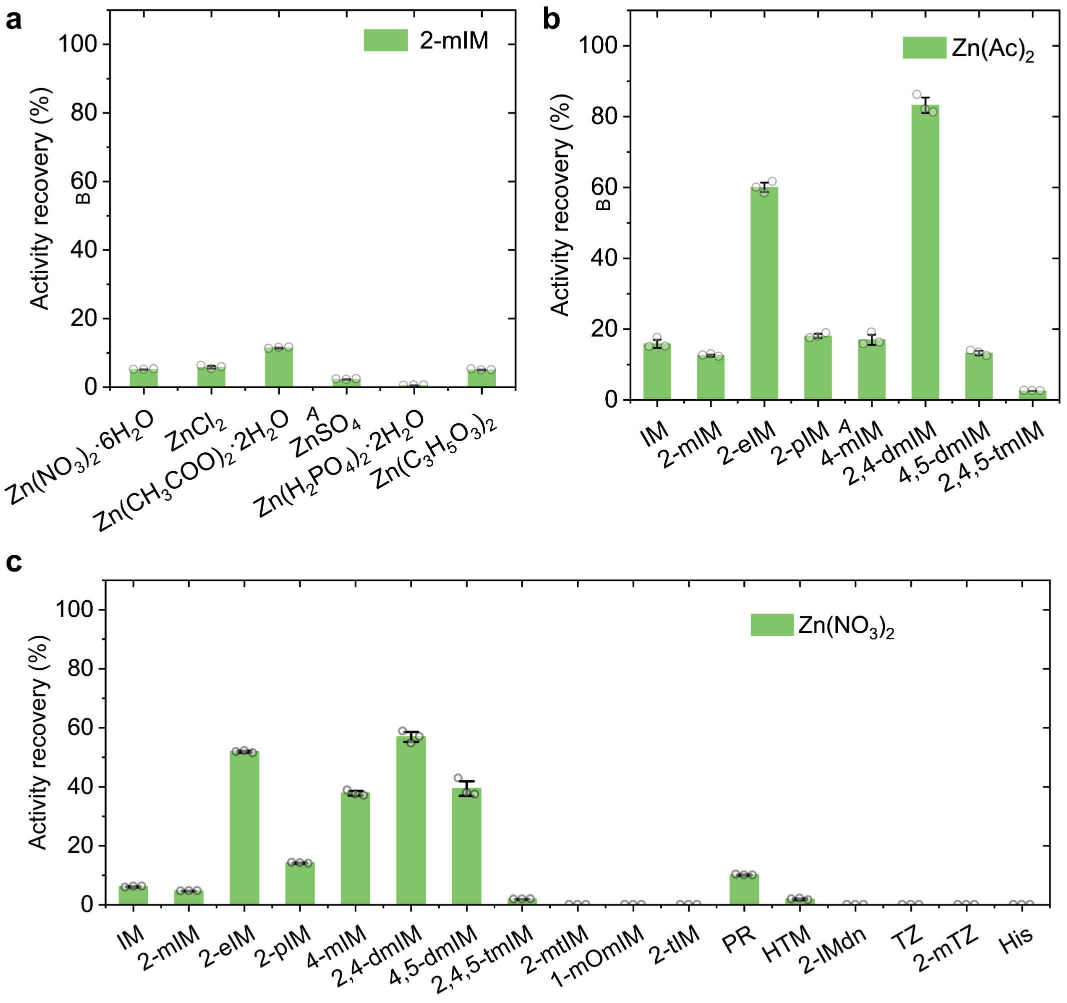

Supplementary Figure 1. First three trials of OVAT optimization. **a** 1.25 M 2-mIM in combination with different zinc salts; **b** 0.31 M Zn(Ac)$_2$ in combination with different ligands; **c** 0.31 M Zn(NO$_3$)$_2$ in combination with different ligands. Data were represented as mean ± SD (*n*=3).

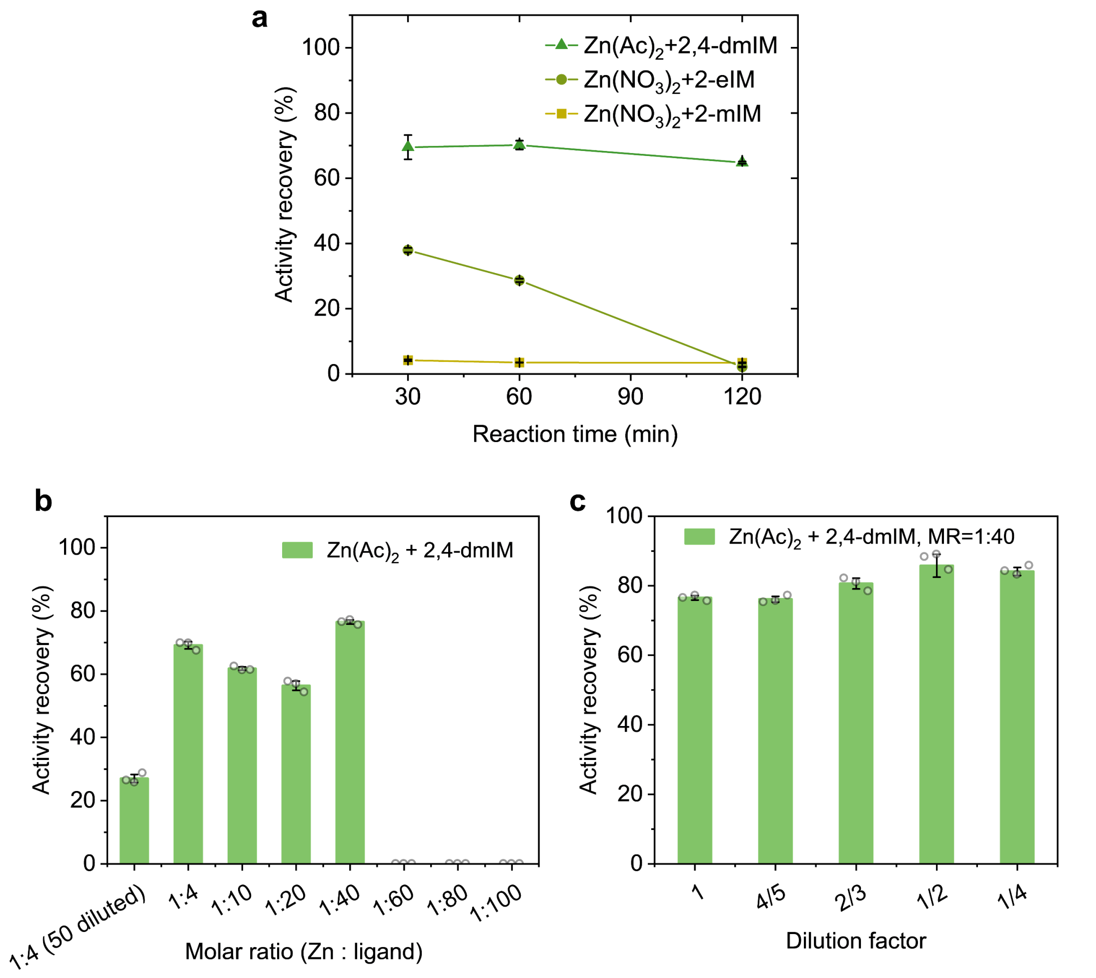

Supplementary Figure 2. Following three trials of OVAT optimization. a Activity recovery of three combinations with different reaction times; **b** Activity recovery of $Zn(Ac)_2$+2,4-dmIM with different molar ratios; **c** Activity recovery of $Zn(Ac)_2$+2,4-dmIM with different dilution factors. Data were represented as mean ± SD (*n*=3).

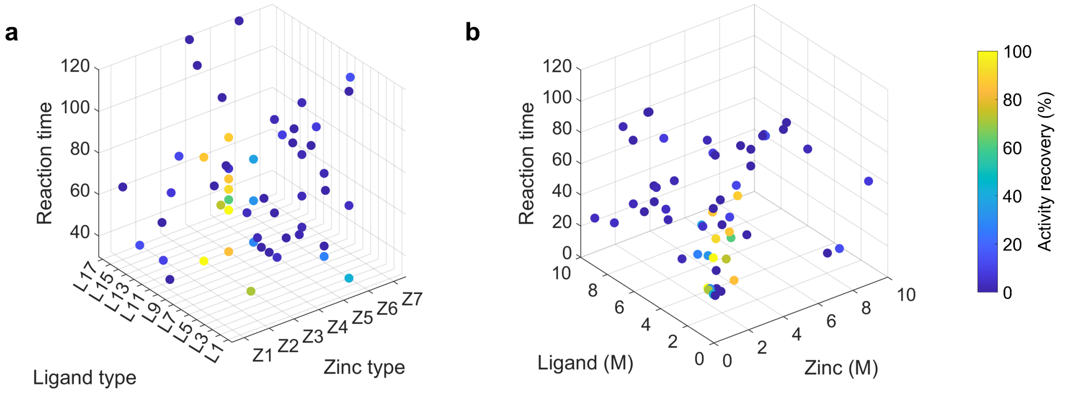

Supplementary Figure 3. Reaction results of GOx by LocalSearch. a Precursor types and **b** concentrations of experiments of GOx by LocalSearch.

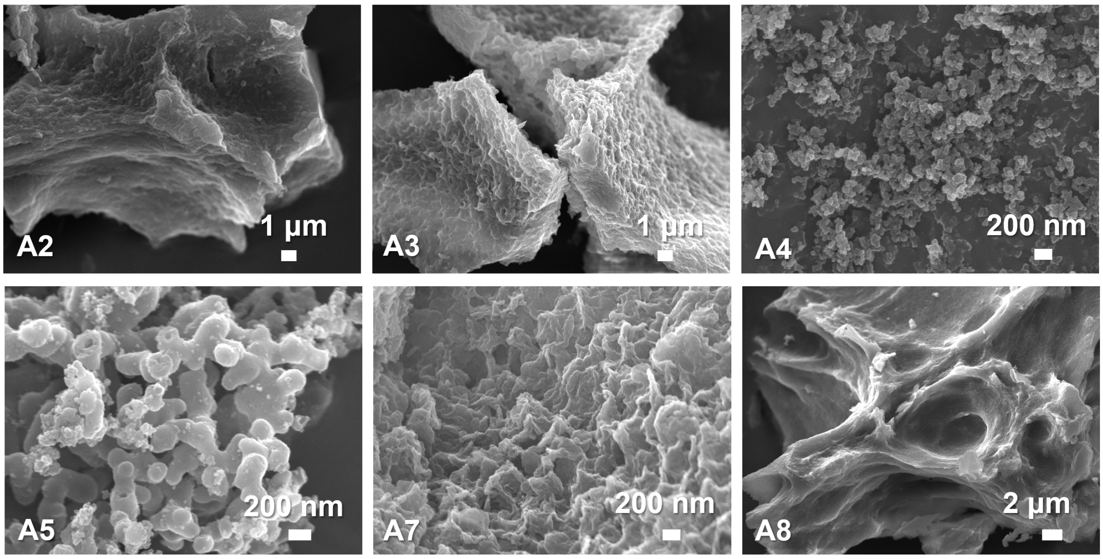

Supplementary Figure 4. Selected SEM images of GOx hybrid biocatalysts with high activity.

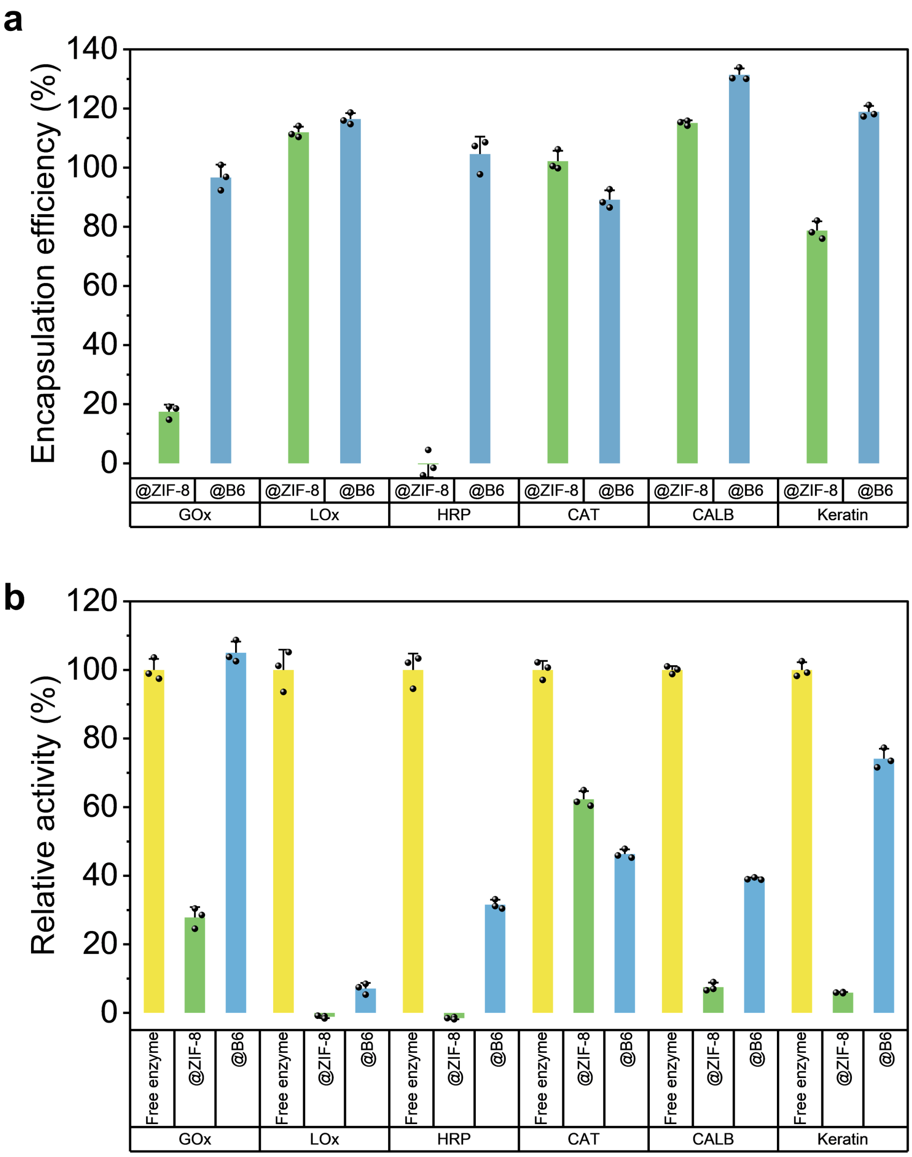

Supplementary Figure 5. Encapsulation of different enzymes using ZIF-8 and B6. a Encapsulation efficiency and **b** relative activity of different enzymes immobilized by ZIF-8 and B6. Data were represented as mean ± SD (n=3).

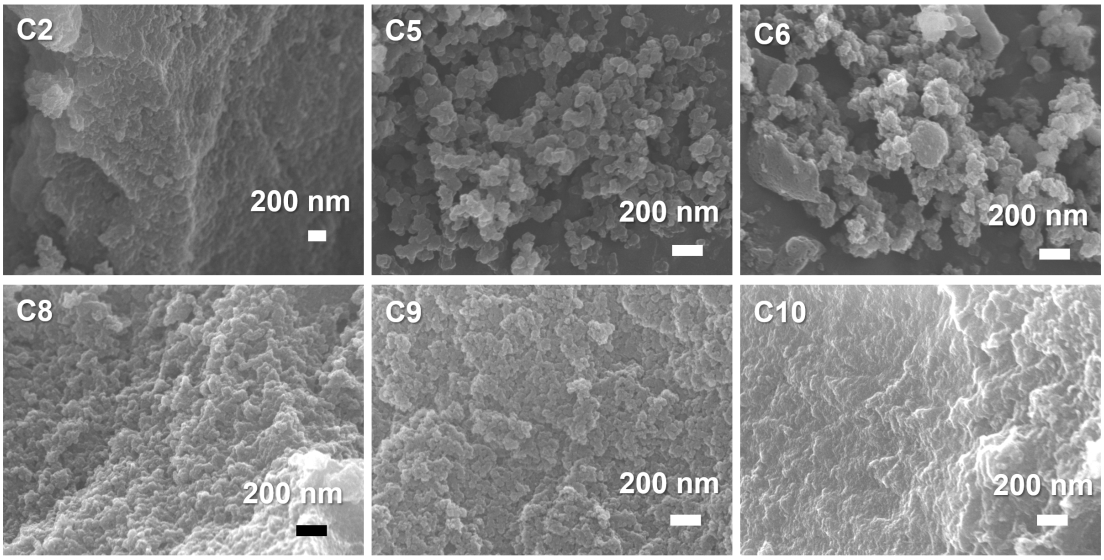

Supplementary Figure 6. Selected SEM images of CAT hybrid biocatalysts with high activity.

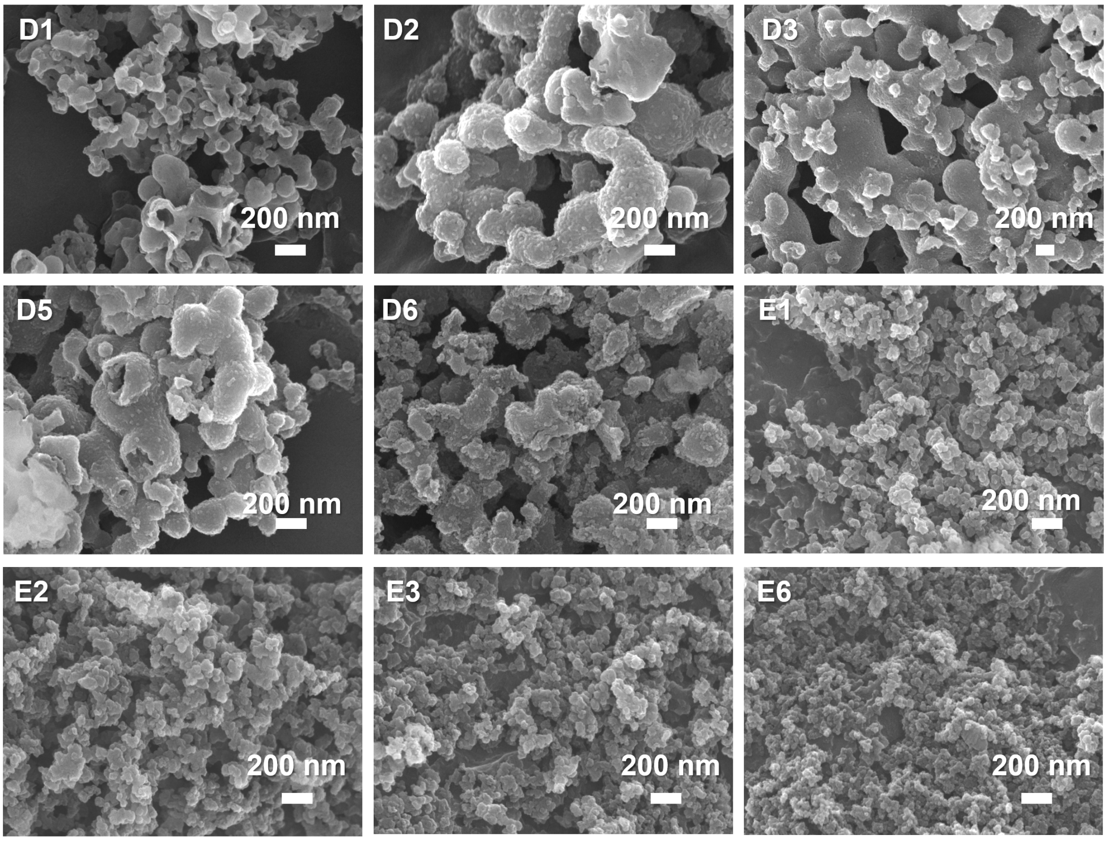

Supplementary Figure 7. Selected SEM images of CALB hybrid biocatalysts with high activity.

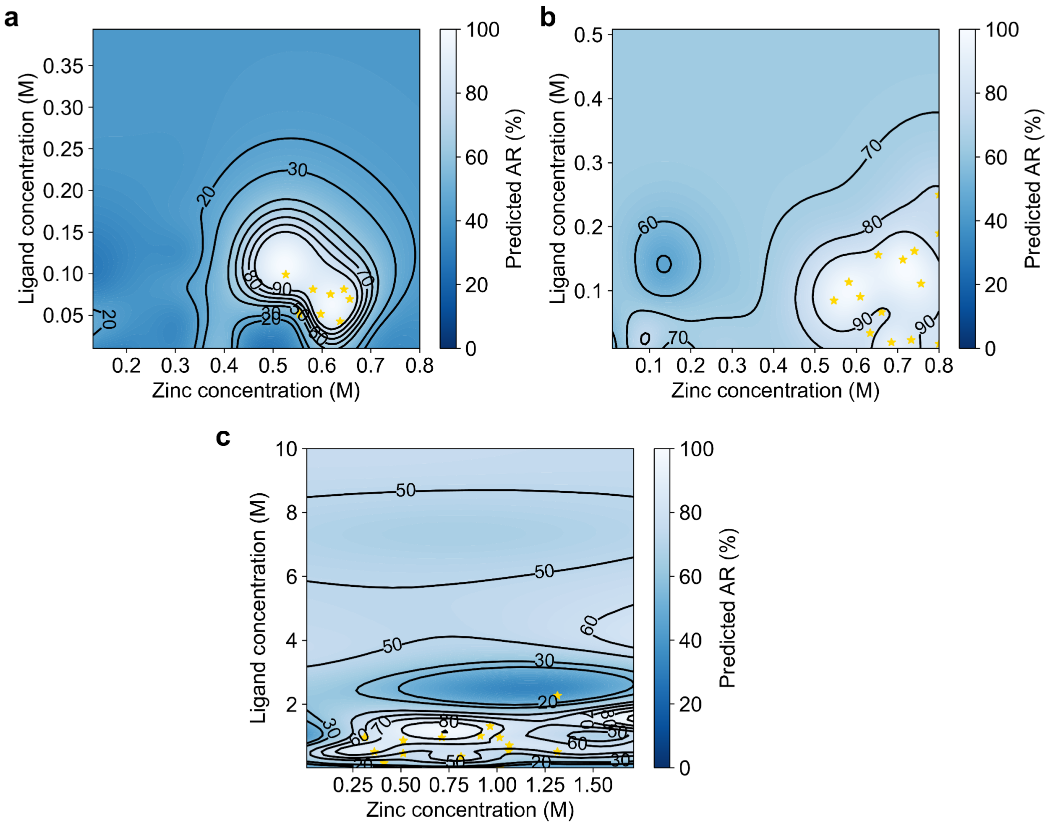

Supplementary Figure 8. Response surface modeling of activity recovery with full range of precursor concentrations. a GOx ($Zn(Ac)_2$ and 2-tIM), **b** CAT ($Zn(Ac)_2$ and HTM), **c** CALB ($Zn(NO_3)_2$ and 2,4-dmIM)

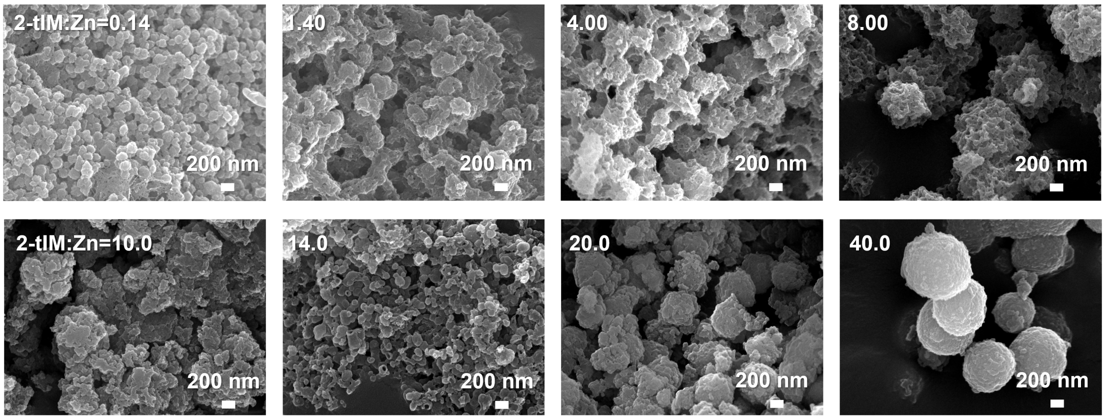

Supplementary Figure 9. SEM images of GOx hybrid biocatalysts synthesized with $Zn(Ac)_2$ and 2-tIM with different molar ratios.

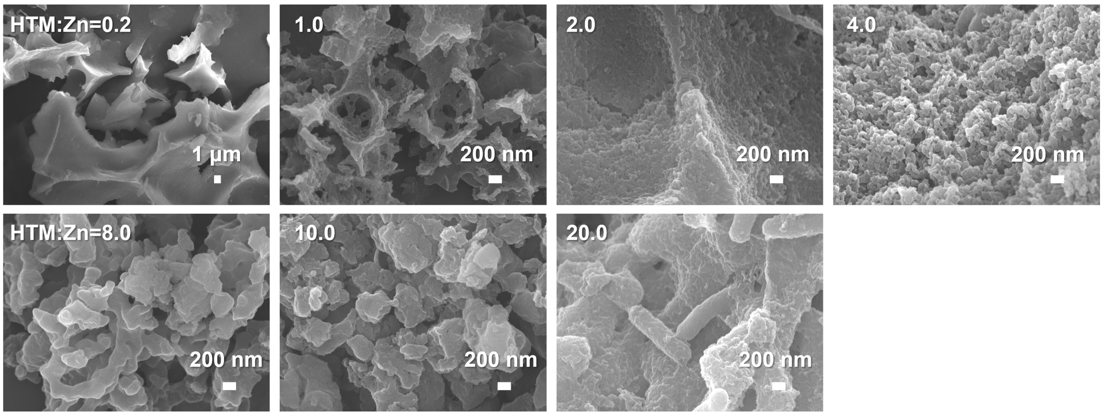

Supplementary Figure 10. SEM images of CAT hybrid biocatalysts synthesized with $Zn(Ac)_2$ and HTM with different molar ratios.

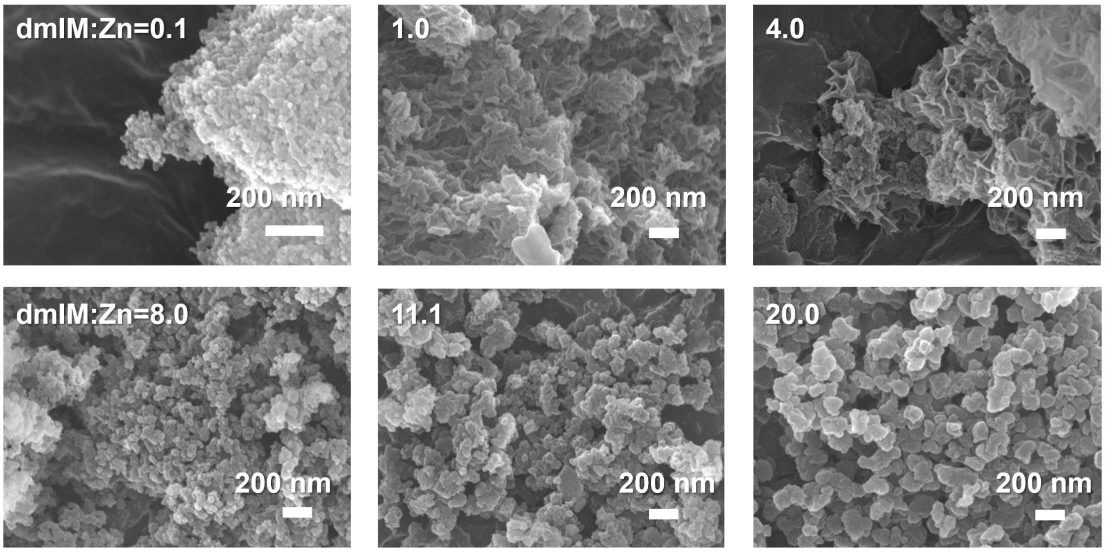

Supplementary Figure 11. SEM images of CALB hybrid biocatalysts synthesized with $Zn(NO_3)_2$ and 2,4-dmIM with different molar ratios.

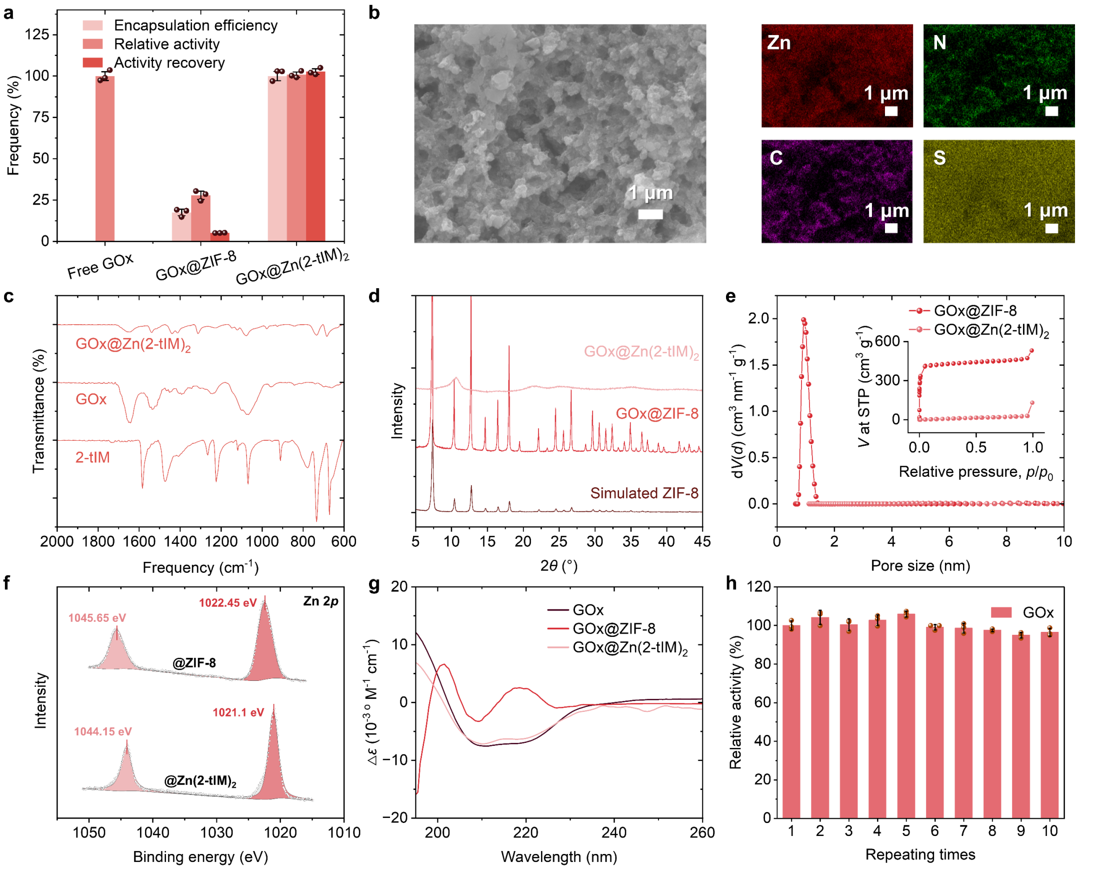

Supplementary Figure 12. Structural characterizations of GOx@Zn(2-tIM)$_2$. a Enzymatic activity of GOx@ZIF-8 and GOx@Zn(2-tIM)$_2$. **b** SEM and EDS mapping of GOx@Zn(2-tIM)$_2$. **c** FTIR curves, **d** XRD curves, **e** pore size distribution and N_2 adsorption and desorption curves (insert), **f** high resolution Zn 2*p* XPS spectra, and **g** CD diagram of GOx@ZIF-8 and GOx@Zn(2-tIM)$_2$. **h** Reuse performance of GOx@Zn(2-tIM)$_2$. Data were represented as mean ± SD (*n*=3).

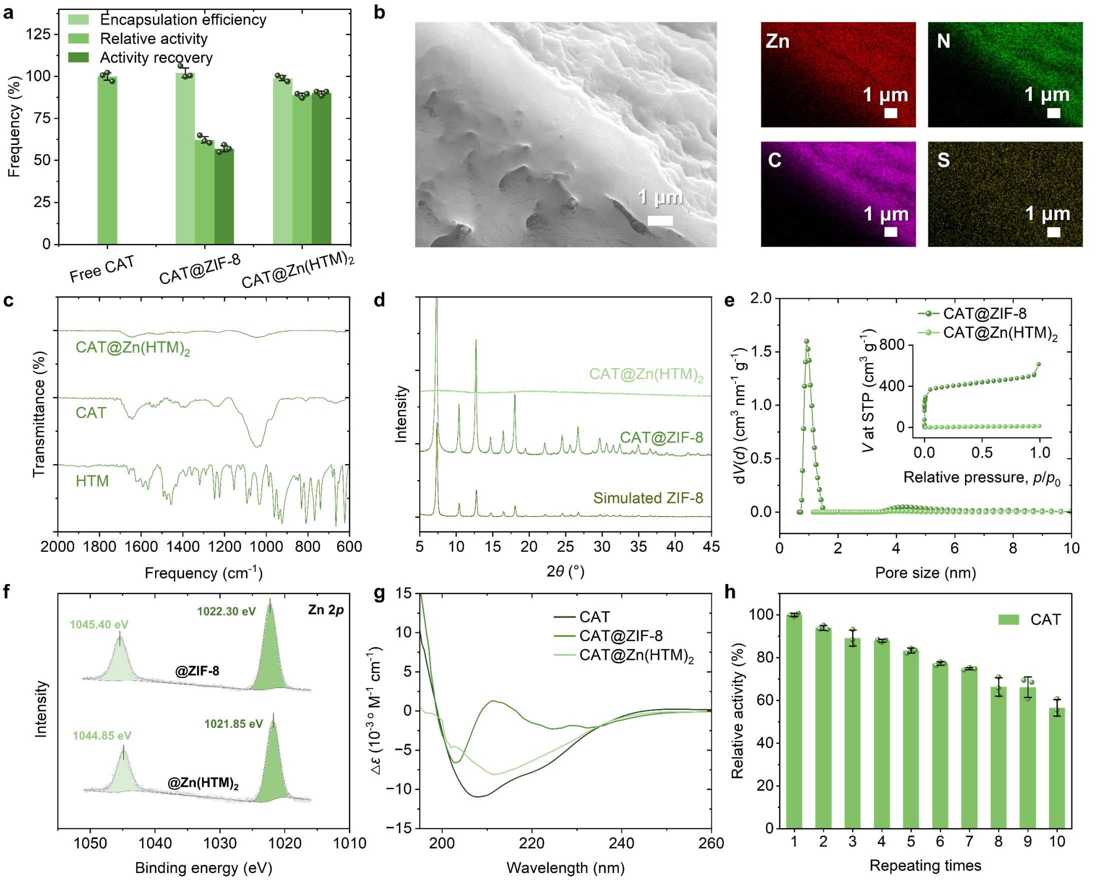

Supplementary Figure 13. Structural characterizations of CAT@Zn(HTM)₂. a Enzymatic activity of CAT@ZIF-8 and CAT@Zn(HTM)₂. **b** SEM and EDS mapping of CAT@Zn(HTM)₂. **c** FTIR curves, **d** XRD curves, **e** pore size distribution and N_2 adsorption and desorption curves (insert), **f** high resolution Zn 2*p* XPS spectra, and **g** CD diagram of CAT@ZIF-8 and CAT@Zn(HTM)₂. **h** Reuse performance of CAT@Zn(HTM)₂. Data were represented as mean ± SD (*n*=3).

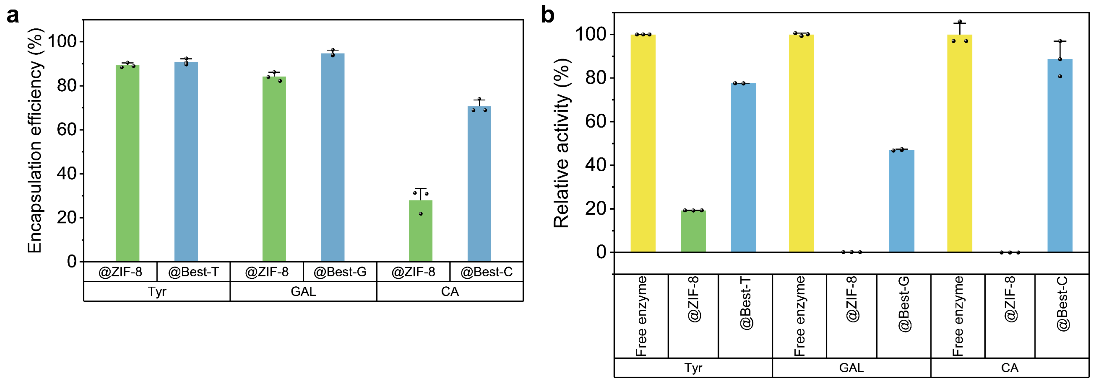

Supplementary Figure 14. Encapsulation of different enzymes immobilized by ZIF-8 and respective best carriers. a Encapsulation efficiency and **b** relative activity of nanohybrids. Data were represented as mean ± SD (*n*=3).

Supplementary Note 5:

Respective best carrier: For each enzyme, PHBO was initiated from ZIF-8 and iteratively screened 8 carriers per round for three rounds. The carrier yielding the highest activity recovery was selected as the "best carrier" and its encapsulation efficiency and relative activity were subsequently measured. Even with limited optimization, substantial improvements were observed in both encapsulation efficiency and relative activity compared with the initial ZIF-8 baseline. Specifically, the relative activity of Tyr, GAL, and CA immobilized with ZIF-8 were 19.29%, 0.16%, and -0.03%, respectively, while those immobilized with their respective best carrier increased to 77.57%, 47.06%, and 88.82%, respectively.

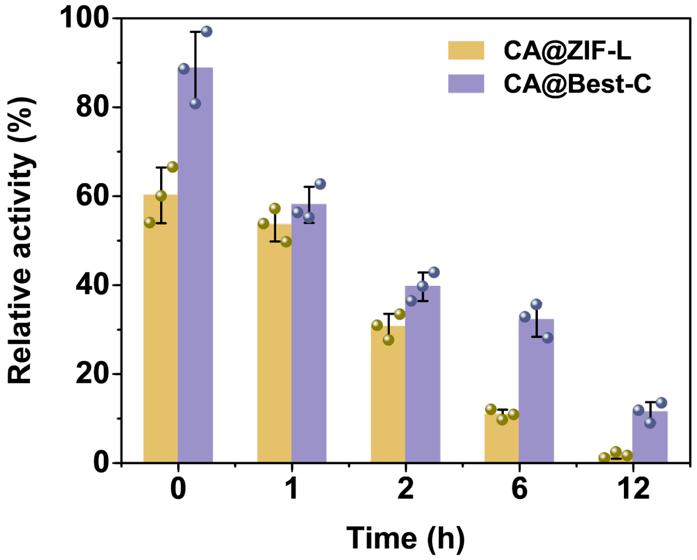

Supplementary Figure 15. Thermal stability of CA immobilized on different carriers in 30% MDEA solution at 80 °C. N-methyldiethanolamine (MDEA) was a widely used organic amine solvent in chemical absorption of CO_2-capture process. Data were represented as mean ± SD (*n*=3).

Supplementary Note 6:

Relative activity was monitored over time to compare the stability of CA immobilized by AI-identified carriers and control formulations. The ZIF-L carrier was chosen as the control[4] and synthesized following the procedure reported in the reference. Under the heated and alkaline condition, CA@ZIF-L rapidly lost activity, whereas the AI-identified best carrier maintained 32.3% activity after 6 h of heating.

Supplementary References:

[1] Cano, A., Acosta, M. & Arnao, M. B. A method to measure antioxidant activity in organic media: application to lipophilic vitamins. *Redox Rep*. **5,** 365-370 (2000).

[2] Nobel, R. W. & Gibson, Q. H. The reaction of ferrous horseradish peroxidase with hydrogen peroxide. *J. Biol. Chem.* **245,** 2409-2413 (1970).

[3] Bessey, O. A. & Love, R. H. Preparation and measurement of the purity of the phosphatase reagent, disodium p-nitrophenyl phosphate. *J. Biol. Chem.* **196,** 175-178 (1952).

[4] Shao, P., et al. Shape controlled ZIF-8 crystals for carbonic anhydrase immobilization to boost CO2 uptake into aqueous MDEA solution. *Sep. Purif. Technol.* **315,** 123683 (2023).
