# Supplementary material for: Accelerated discovery of highly active enzyme nanohybrids with parallelized Bayesian optimization in hybrid space
Source: Nat Commun. 2026 Mar 7;17:3634. doi: 10.1038/s41467-026-70251-3 (PMC13096166; doi:10.1038/s41467-026-70251-3)
Supplement: Supplementary file 2 — Description of Additional Supplementary Files [file 41467_2026_70251_MOESM2_ESM.pdf]

Description of Additional Supplementary Files

File Name: Supplementary Data 1

Description: Raw data from characterizations in Figs and Suppl. Figs.
