# Supplementary material for: Accelerated discovery of highly active enzyme nanohybrids with parallelized Bayesian optimization in hybrid space
Source: Nat Commun. 2026 Mar 7;17:3634. doi: 10.1038/s41467-026-70251-3 (PMC13096166; doi:10.1038/s41467-026-70251-3)
Supplement: Supplementary file 4 — Reporting Summary [file 41467_2026_70251_MOESM4_ESM.pdf]

nature portfolio

Corresponding author(s): Jun Ge, Zhihong Yuan

Last updated by author(s): Feb 16, 2026

Reporting Summary

Nature Portfolio wishes to improve the reproducibility of the work that we publish. This form provides structure for consistency and transparency in reporting. For further information on Nature Portfolio policies, see our Editorial Policies and the Editorial Policy Checklist.

Statistics

For all statistical analyses, confirm that the following items are present in the figure legend, table legend, main text, or Methods section.

n/a	Confirmed	
☐	☒	The exact sample size (*n*) for each experimental group/condition, given as a discrete number and unit of measurement
☐	☒	A statement on whether measurements were taken from distinct samples or whether the same sample was measured repeatedly
☒	☐	The statistical test(s) used AND whether they are one- or two-sided<br>*Only common tests should be described solely by name; describe more complex techniques in the Methods section.*
☒	☐	A description of all covariates tested
☒	☐	A description of any assumptions or corrections, such as tests of normality and adjustment for multiple comparisons
☐	☒	A full description of the statistical parameters including central tendency (e.g. means) or other basic estimates (e.g. regression coefficient) AND variation (e.g. standard deviation) or associated estimates of uncertainty (e.g. confidence intervals)
☒	☐	For null hypothesis testing, the test statistic (e.g. *F*, *t*, *r*) with confidence intervals, effect sizes, degrees of freedom and *P* value noted<br>*Give P values as exact values whenever suitable.*
☐	☒	For Bayesian analysis, information on the choice of priors and Markov chain Monte Carlo settings
☒	☐	For hierarchical and complex designs, identification of the appropriate level for tests and full reporting of outcomes
☒	☐	Estimates of effect sizes (e.g. Cohen's *d*, Pearson's *r*), indicating how they were calculated

Our web collection on statistics for biologists contains articles on many of the points above.

Software and code

Policy information about availability of computer code

Data collection	UV probe; v2.61; Tecan SPARKCONTROL
Data analysis	Origin 2025; MATLAB R2024a; Microsoft Office 365 (Excel, PowerPoint); Python 3; NumPy; Pandas; PyTorch; BoTorch

For manuscripts utilizing custom algorithms or software that are central to the research but not yet described in published literature, software must be made available to editors and reviewers. We strongly encourage code deposition in a community repository (e.g. GitHub). See the Nature Portfolio guidelines for submitting code & software for further information.

Data

Policy information about availability of data

All manuscripts must include a data availability statement. This statement should provide the following information, where applicable:

- Accession codes, unique identifiers, or web links for publicly available datasets
- A description of any restrictions on data availability
- For clinical datasets or third party data, please ensure that the statement adheres to our policy

The data that supports the findings of this study, including datasets generated and analyzed, are provided within this paper and its Supplementary Information files. Source Data are provided with this paper.

Research involving human participants, their data, or biological material

Policy information about studies with human participants or human data. See also policy information about sex, gender (identity/presentation), and sexual orientation and race, ethnicity and racism.

Reporting on sex and gender	N/A
Reporting on race, ethnicity, or other socially relevant groupings	N/A
Population characteristics	N/A
Recruitment	N/A
Ethics oversight	N/A

Note that full information on the approval of the study protocol must also be provided in the manuscript.

Field-specific reporting

Please select the one below that is the best fit for your research. If you are not sure, read the appropriate sections before making your selection.

☐ Life sciences ☐ Behavioural & social sciences ☒ Ecological, evolutionary & environmental sciences

For a reference copy of the document with all sections, see nature.com/documents/nr-reporting-summary-flat.pdf

Ecological, evolutionary & environmental sciences study design

All studies must disclose on these points even when the disclosure is negative.

Study description	This study aimed to optimize enzyme immobilization conditions using a machine learning-driven approach (PHBO). Three enzymes—glucose oxidase (GOx), catalase (CAT), and Candida antarctica lipase B (CALB)—were used as model systems. Workflow: PHBO suggest 8 sets of experimental conditions in parallel based on the prior knowledge database. These 8 experiments were artificially performed to produce 8 kinds of nanohybrids. Then, the activity of nanohybrids were tested, with each sample repeating for 3 times. The mean activity recovery were fed back to the model. PHBO would recommend again in these iterations, until the requirements of researchers were reached.
Research sample	With the help pf PHBO, 88 sets of nanohybrids were synthesized and evaluated for GOx, 56 sets for CAT, and 72 sets for CALB.
Sampling strategy	Parallel experiments independent of each other were used in this study.
Data collection	The data were analyzed by the instrument and then the raw data were copied. The experimental data were recorded by the experimenter in a lab book and an Excel sheet, respectively.
Timing and spatial scale	Each kind of nanohybrids was synthesized for 30-120 min, respectively. For iterations of GOx, CAT, and CALB, the experiments were conducted within 6 months to ensure the consistency.
Data exclusions	No data was excluded.
Reproducibility	All experimental data involving this study were conducted in three parallel experiments to reduce experimental error. For each experiment, a repeat experiment was performed to verify the reproducibility of the results
Randomization	Randomization studies are not applicable to this study.
Blinding	As no clinical trials were involved, blinding was not relevant to this study

Did the study involve field work? ☐ Yes ☒ No

Reporting for specific materials, systems and methods

We require information from authors about some types of materials, experimental systems and methods used in many studies. Here, indicate whether each material, system or method listed is relevant to your study. If you are not sure if a list item applies to your research, read the appropriate section before selecting a response.

Materials & experimental systems

n/a	Involved in the study
☒	☐ Antibodies
☒	☐ Eukaryotic cell lines
☒	☐ Palaeontology and archaeology
☒	☐ Animals and other organisms
☒	☐ Clinical data
☒	☐ Dual use research of concern
☒	☐ Plants

Methods

n/a	Involved in the study
☒	☐ ChIP-seq
☒	☐ Flow cytometry
☒	☐ MRI-based neuroimaging

Plants

Seed stocks	N/A
Novel plant genotypes	N/A
Authentication	N/A
